# Resilience and prosociality: pathways to strengthen teachers' self-efficacy in the classroom

**DOI:** 10.3389/fpsyg.2025.1660879

**Published:** 2025-09-12

**Authors:** Manuel Mieres-Chacaltana, Sonia Salvo-Garrido, Sergio Dominguez-Lara

**Affiliations:** ^1^Departamento de Diversidad y Educación Intercultural, Universidad Católica de Temuco, Temuco, Chile; ^2^Departamento de Matemática y Estadística, Universidad de La Frontera, Temuco, Chile; ^3^Instituto de Investigación FCCTP, Universidad de San Martín de Porres, Lima, Peru

**Keywords:** teacher self-efficacy, resilience, prosocial behavior, classroom management, student engagement, instructional strategies, socioemotional competencies, primary education

## Abstract

This study investigates the relationship between resilience and teacher self-efficacy in Chilean elementary school teachers, considering the mediating role of prosocial behavior. Based on a cross-sectional design and a large national sample (*N* = 1,426), structural equation modeling (SEM) was used to analyze both direct and indirect associations. Results revealed that resilience significantly predicts teacher self-efficacy, both directly and indirectly through prosociality. Among the three self-efficacy dimensions, stronger associations were observed in instructional strategies and student engagement. These findings highlight the importance of strengthening socioemotional competencies—such as resilience and prosocial tendencies—in teacher training programs, as key mechanisms to improve classroom management, teaching effectiveness, and student motivation in educational contexts.

## 1 Introduction

Recent studies highlight that teacher self-efficacy is a critical determinant of professional behavior, influencing emotional regulation, instructional quality, and classroom engagement (Caprara et al., 2005; Wang et al., 2024). Despite growing interest in the factors that enhance self-efficacy, limited attention has been given to the socioemotional pathways that may explain its development in teachers facing complex classroom environments (Davis et al., 2022; Kraft et al., 2020).

Among these socioemotional factors, resilience has been extensively studied as a personal resource that enables individuals to overcome adversity and maintain goal-oriented behavior (Grotberg, 2003; Pozo-Rico et al., 2023). However, resilience alone may not fully account for how teachers translate coping capacity into effective instructional and interpersonal strategies. In this context, prosocial behavior emerges as a key interpersonal disposition that facilitates supportive relationships and cooperative classroom climates (Brass et al., 2024; Kim and Cillessen, 2023)—conditions known to strengthen self-efficacy beliefs (Caprara et al., 2005; Bandura, 2016).

The present study focuses on prosociality as the mediating mechanism between resilience and teacher self-efficacy, based on empirical and theoretical evidence suggesting that prosocial actions activate self-perceptions of competence, particularly in relational and pedagogical domains (Caprara et al., 2005; Kim and Cillessen, 2023). While other emotional variables such as empathy or emotional regulation are relevant, prosociality reflects an enacted behavior that can be directly observed and reinforced within educational contexts (Flores-Piñero et al., 2024; Wentzel, 2022), making it theoretically suitable for modeling mediation.

In 2015, the United Nations adopted a set of universal goals aimed at sustainable development (United Nations, 2016), assigning education a key role in their achievement (Trevors and Saier, 2010; United Nations, 2022). Accordingly, it has promoted equitable access to quality education at all levels. However, UNESCO (2024) has warned of insufficient attention to essential dimensions of learning, particularly social and affective aspects such as students' emotional experiences and feelings, which are often absent from contemporary debates on learning (Howard, 2018). Education for the twenty-first century therefore requires integrating relational and socio-emotional dimensions (Costa and Cipolla, 2025), addressing basic emotional needs to support learning (Hammond, 2015), and moving beyond purely cognitive approaches (Howard et al., 2020).

The educational vision for 2050 positions schools as key agents of economic development and social governance (Kay, 2020), promoting a prosocial orientation that extends across different scales. This orientation refers to a set of dispositions and behaviors aimed at the wellbeing of others (Hart and Hart, 2023). In this regard, prosociality in the teaching profession has shown positive effects on helping behaviors (Aldabbagh et al., 2022; Flores-Piñero et al., 2024; Kim and Cillessen, 2023), sharing (Abramczyk and Jurkowski, 2020; Tan and Kaveri, 2024; Wei et al., 2023), caring (Brass et al., 2024; Chen et al., 2024; Wentzel, 2022) and empathy (Hong et al., 2022; Samavi et al., 2022; Yang and Zhang, 2024), which is considered an essential component in adulthood (Caprara et al., 2005). These practices also benefit teachers, promoting their wellbeing (Corrente et al., 2022; Kim et al., 2022), engagement (Soininen et al., 2023), job satisfaction (Aydin Sunbul and Gordesli, 2021; Reeves et al., 2017), teaching quality and professional development (Akiba and Liang, 2016; Gore et al., 2017; Jurkowski and Abramczyk, 2024), as well as mitigating work-related stress (Paliliunas et al., 2024), a critical factor in the current teacher availability crisis (Steiner and Woo, 2021).

In addition, new challenges require resilient teachers who can face and adapt to adversity (Moll Riquelme et al., 2022; Sisto et al., 2019). Resilience, which is activated under challenging situations (Grotberg, 2003), is particularly valuable in today's uncertain environment (Bravo-Sanzana et al., 2023). Since all individuals can develop resilience—even in complex contexts (Grotberg, 2001, 2003)—the education system plays a decisive role in its promotion. Teaching how to manage emotions and concerns is thus a fundamental teaching competency.

The literature has documented positive links between resilience and prosociality (Liu and Ngai, 2019; Silveira et al., 2022; Villalta and Saavedra, 2011; Xiang et al., 2023), particularly in educational responses to students at social or academic risk, through support networks such as family and peers (Alhawsawi et al., 2022; Edmonds et al., 2022; Escalante Mateos et al., 2021; Salinas-Falquez et al., 2022). Teacher resilience is also associated with wellbeing (Pozo-Rico et al., 2023), health (Salvo et al., 2017), motivation, and improved student performance (Cachón Zagalaz et al., 2020; Wang and Lo, 2022; Yang and Wang, 2022). Furthermore, social factors have a greater impact than individual ones in the development of resilience (Ainsworth and Oldfield, 2019; Cann et al., 2024; Durrani and Makhmetova, 2025; Hartcher et al., 2023).

This highlights the urgency of creating school environments that promote emotional and resilient learning (Beltman et al., 2011; Johnson and Down, 2013; Pozo-Rico et al., 2023; Ungar, 2012), which requires consideration of teachers' agency, values, talents, and levels of self-efficacy (Bandura, 1995; Duan et al., 2024). Teacher self-efficacy, defined here as the belief in one's ability to manage the classroom, create environments conducive to learning, and implement instructional strategies that support all students' learning, plays a key role. From the perspective of social cognitive theory, individuals act according to their perceived competence, setting goals, regulating behavior, and evaluating performance (Bandura, 1986, 1997). In this framework, self-efficacy is a central mechanism of human agency (Bandura, 1986, 1997, 2001), influencing self-regulation, effort, perseverance, and emotional management (Caprara et al., 2005; Bandura, 2016). It also predicts performance through its effects on motivation, engagement, and persistence in the face of difficulties (Bandura, 1997, 2016).

In the teaching domain, self-efficacy refers to the belief in one's ability to positively influence student learning and behavior (Collie et al., 2012). Teacher self-efficacy, in particular, enables the creation of classroom climates conducive to educational achievement. Research has established its relevance in education (Barbaranelli et al., 2019; Di Giunta et al., 2013; Diseth et al., 2012; Mohamadi et al., 2011), highlighting it as a predictor of prosocial behavior (Davis et al., 2022; Eisenberg et al., 2002; Gómez Tabares, 2018; Liu and Ngai, 2019) and as a factor associated with resilience in adverse situations (Kraft et al., 2020; Mansfield et al., 2012).

In conclusion, in the face of twenty-first-century challenges, promoting teachers' social self-efficacy is essential for advancing inclusion (Mudhar et al., 2023) and strengthening collaborative pedagogy (Al-Samarraie et al., 2020; Nielsen, 2023; Wang, 2024). Resilience, prosociality, and self-efficacy are interrelated dimensions that mutually reinforce one another (Mieres-Chacaltana et al., 2025; Salvo-Garrido et al., 2024). Thus, a resilient condition, mediated by prosociality, can enhance teachers' social self-efficacy, improving classroom management and fostering active student participation.

Based on these theoretical premises and the previously cited findings, the objective of this study was to evaluate a conceptual model in which resilience serves as the foundation of teacher self-efficacy through both a direct relationship and an indirect one mediated by prosociality. The research questions guiding the study were: how do the foundational or structural components of resilience influence teacher self-efficacy? What mediating effect does prosociality have on the relationship between resilience and teacher self-efficacy? Hypothesis 1 (H1) posits that resilience constitutes a foundational condition for the expression of teacher self-efficacy, and therefore, a direct positive effect is expected (H1). Hypothesis 2 (H2) posits that prosociality also contributes to teacher self-efficacy by mediating its relationship with resilience.

## 2 Methods

### 2.1 Participants

The target population of this study comprised elementary school teachers working in public and government-subsidized schools in Chile (*N* = 85,298). A stratified multistage probabilistic sampling design was originally planned, considering region, area of residence (urban/rural), type of school funding, and gender as stratification variables. Based on this design, a theoretical sample size was calculated using a 95% confidence level, a 2.5% margin of error, and maximum variance (*p* = *q* = 0.5).

However, as participation was voluntary, data collection was ultimately carried out through an open invitation to schools that met the inclusion criteria across macrozones. Priority was given to schools with at least 10 teachers in the first cycle of elementary education to ensure respondent anonymity. Therefore, although the sampling was theoretically stratified, the final sample is best described as a non-probabilistic, self-selected sample that nonetheless preserved representation criteria across key educational strata.

Teachers accessed the study through a digital link that included informed consent, a sociodemographic questionnaire, and the psychometric instruments in self-administered format. Ethical approval was granted by the Scientific Ethics Committee of the Universidad de La Frontera (Act N°119_22).

The final sample consisted of 1,426 teachers (1.67% of the target population), ranging in age from 21 to 70 years (M = 41.5; SD = 10.8). Of the total, 77.3% identified as women and 22.7% as men. Regarding school characteristics, 81.2% were located in urban areas, 83.6% were publicly funded, and 16.4% were government-subsidized private schools. Teaching experience ranged from < 1 year to 48 years (M = 14.2; SD = 10.1). All participants were actively teaching in classrooms at the time of data collection.

### 2.2 Measures

Resilience Scale for Youth and Adults (SV-RES60). This scale was developed in the Chilean population (Saavedra and Villalta, 2008) and validated among elementary school teachers (Salvo-Garrido et al., 2023). It measures a general resilience factor and 12 residual factors. For this study, an abbreviated version was used, consisting of 15 items distributed across three structural dimensions of resilience: “I am,” “I have,” and “I can,” based on Grotberg's (1995) theoretical framework. The psychometric analysis conducted on the current sample using a bifactor model within the framework of exploratory structural equation modeling (ESEM) demonstrated good fit indices (RMSEA = 0.068; SRMR = 0.015; CFI = 0.985; TLI = 0.969) and high reliability (α = 0.927; ω = 0.958).

Adult Prosocialness Behavior Scale (APBS). This is a 16-item self-report instrument developed by Caprara et al. (2005), and validated in Chile with teachers (Mieres-Chacaltana et al., 2023) and pre-service teacher education students (Mieres-Chacaltana et al., 2020). It assesses prosocial behavior through a unidimensional model with four residual factors: helping, sharing, caring, and empathizing. In the present study, ESEM analysis confirmed this structure with a dominant general factor and four specific factors. The model showed excellent fit (RMSEA = 0.044; SRMR = 0.012; CFI = 0.995; TLI = 0.987) and high reliability (α = 0.931; ω = 0.962).

Teachers' Sense of Efficacy Scale (TSES). Developed by Tschannen-Moran and Woolfolk Hoy (2001) and validated with a sample of Chilean elementary school teachers (Gálvez-Nieto et al., 2023). The scale consists of 24 items rated on a five-point ordinal Likert scale, grouped into three dimensions: classroom management, student engagement, and instructional strategies. In this study, a bifactor model estimated via ESEM confirmed the instrument's structure, showing good fit (RMSEA = 0.068; SRMR = 0.015; CFI = 0.988; TLI = 0.982) and high overall reliability (α = 0.971; ω = 0.974). Subscale reliabilities were also high: efficacy in student engagement (α = 0.971; ω = 0.945), instructional strategies (α = 0.971; ω = 0.962), and classroom management (α = 0.937; ω = 0.957).

### 2.3 Procedure

Data collection was conducted through a coordinated process involving school principals and local educational authorities, recognizing that participating institutions operate under the administrative supervision of the Chilean Ministry of Education. In the initial stage, institutional emails were sent to present the study's objectives and request the collaboration of school leadership teams. These communications emphasized the academic nature of the research and its alignment with current educational priorities.

Subsequently, on-site visits were carried out to provide detailed information about the study, address potential concerns, and facilitate voluntary teacher participation. Teachers who agreed to participate accessed a secure digital link containing the informed consent form, a sociodemographic questionnaire, and the study instruments.

Participation was strictly voluntary and anonymous. No personal identifiers were collected, and responses were processed in aggregate form to ensure confidentiality and reduce the risk of social desirability bias. Data collection was conducted entirely online using the QuestionPro platform in a self-administered format.

This study was approved by the Scientific Ethics Committee of the Universidad de La Frontera (Case No. 053_21; Protocol No. 019/21), in compliance with the ethical standards for research involving human participants, as established in the Declaration of Helsinki and Chilean regulations on data protection in educational research.

### 2.4 Analytical approach

The primary analysis was conducted using structural equation modeling (SEM) with Mplus version 8.4 (Muthén and Muthén, 2017, 2019). The WLSMV (Weighted Least Squares Mean and Variance adjusted) estimator was employed (Satorra and Bentler, 1994), which is appropriate for ordinal variables (Bagheri and Saadati, 2021) and large samples (Bovaird and Koziol, 2012), as it does not require normality assumptions (Li, 2016) and yields more accurate estimates when handling asymmetric data (Li, 2014).

Prior to estimating the structural model, univariate normality of the items was assessed using conventional criteria (skewness < 2; kurtosis < 7; Finney and DiStefano, 2013; Schumacher and Lomax, 1996).

To assess the potential impact of common method bias, Harman's single-factor test was conducted by including all items from the three scales in an unrotated exploratory factor analysis (Podsakoff et al., 2003). The results showed that the first factor accounted for 31.5% of the total variance, which is well below the recommended threshold of 50%. Therefore, common method bias is unlikely to significantly affect the study results.

Model fit was evaluated using multiple indices: the Root Mean Square Error of Approximation (RMSEA), with acceptable values below 0.08 and a 90% confidence interval upper bound below 0.08 (Browne and Cudeck, 1992; Gouveia et al., 2018; Wang and Wang, 2020); and the Standardized Root Mean Square Residual (SRMR), with optimal values below 0.08 and acceptable values below 0.10 (Hu and Bentler, 1999; Kline, 2005).

## 3 Results

Table 1 presents the main descriptive statistics for each scale, including measures of central tendency, dispersion, skewness (g1), and kurtosis (g2).

**Table 1:** Descriptive statistics based on items by scale.

**Scale**	**Min**	**Max**	***M***	**SD**	**g_1_**	**g_2_**
Resilience	15	75	65.6	8.8	−2.1	7.3
Prosociality	16	80	66.5	9.9	−1.3	2.6
Efficacy in classroom management	8	40	32.1	5.6	−0.5	−0.1
Efficacy in student engagement	8	40	32.7	5.3	−0.6	0.1
Efficacy in instructional strategies	8	40	32.9	5.5	–‘0.7	0.2

*M*, Mean; SD, Standard Deviation; g1, Skewness; g2, Kurtosis.

The structural equation model showed a good fit to the data: χ^2^ = 6691.8, df = 1420, *p* < 0.001; χ^2^/df = 4.712; CFI = 0.961; TLI = 0.959; RMSEA = 0.051, 90% CI [.050–0.052]; SRMR = 0.049. These indices indicate that the proposed theoretical model fits the observed data satisfactorily.

All observed variables loaded significantly and positively onto their respective latent constructs, with high standardized factor loadings, supporting the convergent validity of the factors. Table 2 displays these loadings, organized by latent construct and numbered according to their original order within each scale.

**Table 2:** Standardized factor loadings of observed variables on latent constructs.

**Latent construct**	**Observed variable**	**Factor loading**	**Latent construct**	**Observed variable**	**Factor loading**
Resilience	Item 1	0.797	Efficacy in classroom management	Item 3	0.802
	Item 2	0.708		Item 5	0.875
	Item 3	0.794		Item 8	0.882
	Item 4	0.761		Item 13	0.877
	Item 5	0.789		Item 15	0.863
	Item 21	0.745		Item 16	0.909
	Item 22	0.684		Item 19	0.821
	Item 23	0.794		Item 21	0.835
	Item 24	0.817	Efficacy in student engagement	Item 1	0.743
	Item 25	0.861		Item 2	0.746
	Item 41	0.699		Item 4	0.867
	Item 42	0.786		Item 6	0.889
	Item 43	0.699		Item 9	0.901
	Item 44	0.806		Item 12	0.851
	Item 45	0.865		Item 14	0.849
Prosociality	Item 1	0.757	Efficacy in instructional strategies	Item 22	0.742
	Item 2	0.731		Item 7	0.841
	Item 3	0.822		Item 10	0.901
	Item 4	0.690		Item 11	0.885
	Item 5	0.849		Item 17	0.877
	Item 6	0.782		Item 18	0.826
	Item 7	0.758		Item 20	0.870
	Item 8	0.687		Item 23	0.886
	Item 9	0.816		Item 24	0.879
	Item 10	0.771			
	Item 11	0.530			
	Item 12	0.801			
	Item 13	0.854			
	Item 14	0.741			
	Item 15	0.757			
	Item 16	0.731			

All factor loadings are standardized and statistically significant at *p* < 0.001.

The model explained a substantial proportion of the variance in the three dimensions of teacher self-efficacy: Efficacy in Classroom Management (18.7%), Efficacy in Student Engagement (23%), and Efficacy in Instructional Strategies (17.7%). It also explained 14.1% of the variance in prosociality. All associations were statistically significant (*p* < 0.001).

**Figure 1:**
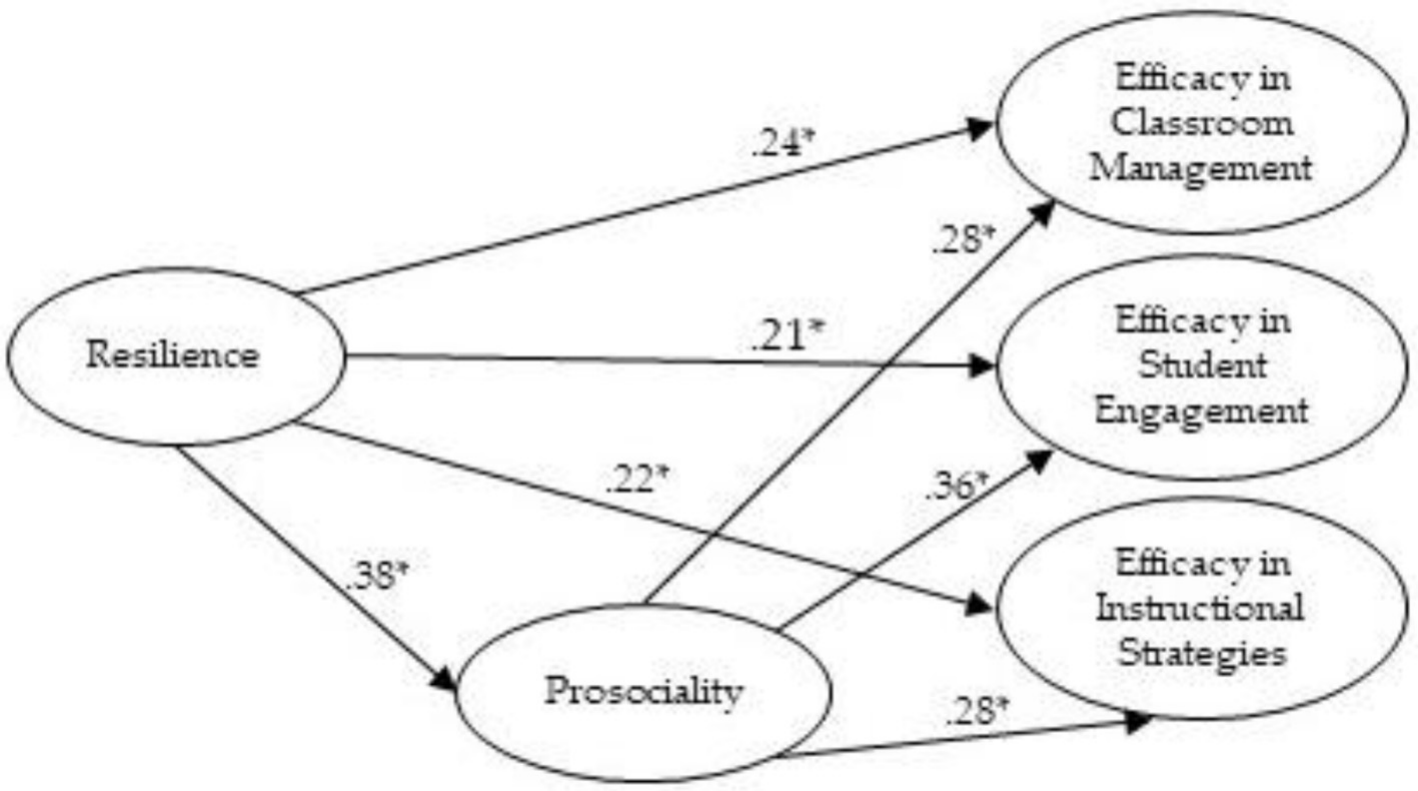
Standardized path diagram illustrating the direct and mediated effects of resilience on teacher self-efficacy via prosociality. All reported paths are significant (*: *p* < 0.001).

Figure 1 graphically summarizes the structural equation model tested in this study. Resilience emerged as a significant positive predictor of all three dimensions of teacher self-efficacy, providing empirical support for Hypothesis 1. Additionally, prosociality acted as a mediator in the relationship between resilience and self-efficacy dimensions, supporting Hypothesis 2. All standardized path coefficients were statistically significant (*p* < 0.001).

Additionally, significant indirect effects of resilience on the three dimensions of teacher self-efficacy were identified, mediated by prosociality, thus supporting Hypothesis 2. Table 3 presents the standardized direct, indirect, and total effects for each outcome variable. These results confirm both the direct influence of resilience and the mediating role of prosociality in shaping teacher self-efficacy beliefs.

**Table 3:** Standardized direct, indirect, and total effects of the resilience and prosociality on self-efficacy.

**Scale**	**Efficacy in classroom management**			**Efficacy in student engagement**			**Efficacy in instructional strategies**		
	**Direct**	**Indirect**	**Total**	**Direct**	**Indirect**	**Total**	**Direct**	**Indirect**	**Total**
Resilience	0.24^*^	0.11^*^	0.35^*^	0.21^*^	0.14^*^	0.35^*^	0.22^*^	0.11^*^	0.33^*^
Prosociality	0.28^*^	-	-	0.36^*^	-	-	0.28^*^	-	-

* = *p* < 0.001.

## 4 Discussion

The main objective of this study was to analyze the effect of resilience on teacher self-efficacy, both directly and indirectly through the mediating role of prosociality, in a sample of teachers working in Chilean public elementary schools. This research responds to the need to generate knowledge that explores alternatives aimed at strengthening socioemotional teaching as a key foundation for 21st-century education (Trevors and Saier, 2010; United Nations, 2016, 2022). These dimensions are essential in the paradigmatic transformation toward an educational model that fosters a more integrated, supportive, and sustainable society in the context of interdependent global challenges (Costa and Cipolla, 2025; Hammond, 2015; Howard, 2018; Howard et al., 2020). However, the persistent omission of social and affective components in teaching practice has called into question the role of education in advancing sustainability (UNESCO, 2024). This study aligns with the priorities set by international education agendas, which increasingly emphasize the integration of socioemotional development in teacher training and the transformation of school cultures to meet the demands of contemporary society.

The first hypothesis proposed that resilience would predict teacher self-efficacy (H1). Regarding the link between resilience and efficacy in classroom management (β = 0.24; *p* < 0.001), the results suggest the importance of fostering teachers' personal resilience, which ultimately translates into increased pedagogical self-efficacy (Ainsworth and Oldfield, 2019; Durrani and Makhmetova, 2025; Escalante Mateos et al., 2021). Classroom management is crucial for creating structured, safe, and emotionally stimulating learning environments, which directly impact students' academic performance and socioemotional wellbeing.

The observed relationship between resilience and efficacy in instructional strategies (β = 0.22; *p* < 0.001) further reinforces the argument that teacher self-efficacy plays a critical role in activating high-quality pedagogical processes. This finding also confirms the enabling potential of personal influence in teaching, fostering self-development, sustained learning, and perseverance in the face of adversity (Bandura, 2016; Wang and Lo, 2022; Yang and Wang, 2022).

This is also reflected in the correlation between resilience and efficacy in student engagement (β = 0.21; *p* < 0.001), reinforcing the notion that resilience directly influences the quality of the teacher-student relationship. This relationship suggests that higher self-efficacy in this specific dimension is associated with greater confidence in the teacher's ability to create emotionally safe and motivating classroom environments. These are key to fostering educational contexts that support student behaviors aligned with personal and social development (Bandura, 1986), as they affect students' intrinsic motivation and willingness to engage actively in their learning processes (Cachón Zagalaz et al., 2020). From this perspective, positive vicarious experiences—such as those generated when teachers serve as significant role models—acquire central formative value, generating a multiplying effect within school culture (Bandura, 1997). These experiences help build more inclusive, horizontal, and participatory educational communities, where all students—regardless of background—feel invited to participate (Mudhar et al., 2023). This becomes even more relevant in contexts of apathy or disengagement, where teachers with high levels of self-efficacy tend to persist, adapt their practice, and explore diverse strategies to engage every student.

Then, the findings of this study support this hypothesis, confirming the positive influence of resilience on all three dimensions of teacher self-efficacy, in line with previous research (Salvo-Garrido et al., 2024; Mieres-Chacaltana et al., 2025), also is consistent with recent evidence by Wang et al. (2024), teacher self-efficacy is positively associated with wellbeing, and this relationship is partially mediated by resilience and teaching satisfaction, highlighting the importance of nurturing socioemotional resources within educational systems. Thus, under this view, the contemporary scenario—marked by complex demands on the teaching profession (Beltman et al., 2011; Bravo-Sanzana et al., 2023; Moll Riquelme et al., 2022; Sisto et al., 2019; Steiner and Woo, 2021)—represents not only a challenge but also an opportunity to strengthen resilience, which precisely emerges in highly complex contexts (Grotberg, 2001, 2003).

More specifically, the efficacy in classroom management is especially relevant in contexts of social vulnerability (Edmonds et al., 2022), underserved rural areas (Wang and Lo, 2022) or among students with special educational needs (Salinas-Falquez et al., 2022). Besides, the perceived efficacy in using instructional strategies extends beyond technical competence to the intentional design of complex learning environments that promote deep and meaningful learning. Thus, teacher resilience, by catalyzing efficacy in instructional strategies, helps consolidate transformative educational practices oriented toward the holistic development of students in increasingly demanding and dynamic contexts. Finally, the efficacy in student engagement is particularly significant in the current global context, where schools are called to play a key role in building active citizenship and promoting more democratic and participatory governance (Kay, 2020). Given the influence of school culture on the development of attitudes, dispositions, and values in individuals (Duan et al., 2024), teacher self-efficacy focused on student engagement constitutes a structural condition for fostering collaborative learning and consolidating more equitable and inclusive educational communities in times of change (Bandura, 1995). It enables individuals to shape the physical and social environments—in this case, schools—through which they exert some control over their lives (Bandura, 2016).

In this sense, this pedagogical orientation aligns with a concept of resilience that transcends individually centered explanations and should instead be understood as a relational, socially situated process embedded within institutional culture (Ungar, 2012). From this perspective, resilience emerges as a quality arising from structurally enabling environments that activate collective processes of containment, adaptation, and transformation (Cann et al., 2024; Hartcher et al., 2023). School culture plays a critical role in shaping such environments, fostering a sense of belonging and support that strengthens teachers' capacity to act prosociality and feel efficacious in their professional roles. Consequently, institutional contexts that promote collaborative norms and emotional sustainability not only reinforce resilience, but also enhance teachers' confidence in their ability to manage classrooms, engage students, and implement instructional strategies effectively.

The second hypothesis proposed that prosociality contributes to the development of teacher self-efficacy by playing a mediating role in its relationship with resilience (H2). The results support this hypothesis, confirming the mediating role of prosociality across all three dimensions of self-efficacy. In this context, the influence of resilience on self-efficacy, mediated by prosociality, enables teachers to perceive themselves as capable of facing the challenges and responsibilities inherent to their professional roles. They also reaffirm the positive association between prosociality and self-efficacy, previously documented from other perspectives (Davis et al., 2022; Eisenberg et al., 2002; Gómez Tabares, 2018; Liu and Ngai, 2019).

From a dimension-specific analytical perspective, in classroom management efficacy, resilience exhibits a moderate direct effect (0.24, *p* < 0.001) complemented by an additional indirect effect (0.11, *p* < 0.001), resulting in a total effect of 0.35 (*p* < 0.001). This indicates that its influence operates both immediately and through the mediating role of prosociality. In turn, prosociality shows a direct effect of 0.28 (*p* < 0.001), slightly higher than the direct effect of resilience, suggesting that prosocial behaviors exert a direct and significant impact on teachers' perceptions of their ability to effectively organize and manage the classroom.

Regarding student engagement efficacy, resilience demonstrates a direct effect of 0.21 (*p* < 0.001) and an indirect effect of 0.14 (*p* < 0.001), yielding a total effect of 0.35 (*p* < 0.001). Although the direct effect is slightly lower than in classroom management, the indirect contribution is greater, indicating that part of its impact is channeled through an intermediate process mediated by prosociality. Moreover, this construct exhibits its strongest direct influence across the entire table (0.36, *p* < 0.001), even surpassing the total effect of resilience, underscoring its importance in promoting students' involvement and active participation.

With respect to instructional strategies efficacy, resilience records a direct effect of 0.22 (*p* < 0.001) and an indirect effect of 0.11 (*p* < 0.001), leading to a total effect of 0.33 (*p* < 0.001) —the lowest among the three dimensions, yet following a consistent pattern of mixed influence. This suggests that teachers' confidence in their ability to implement effective teaching strategies is enhanced both by personal resilience and by mediated processes. Prosociality contributes a direct effect of 0.28 (*p* < 0.001), exceeding the direct effect of resilience in this dimension, indicating that the willingness to act altruistically and cooperatively also strengthens teaching efficacy in the use of resources and instructional methodologies.

The results of H1 and H2 call for the explicit inclusion of socioemotional competencies—such as resilience and prosocial behavior—within national teaching standards, curricular guidelines, and formative assessment systems. Doing so would ensure that emotional and relational dimensions are positioned as core elements of teaching quality and educational equity, rather than treated as peripheral or secondary (UNESCO, 2024; United Nations, 2022). Moreover, integrating these competencies into teacher evaluation and professional development policies would strengthen school systems' capacity to respond effectively to the complex challenges of contemporary education (Beltman et al., 2011; Steiner and Woo, 2021). The aim should be to prepare future professionals who are more willing and able to help (Aldabbagh et al., 2022; Flores-Piñero et al., 2024; Kim and Cillessen, 2023), share (Abramczyk and Jurkowski, 2020; Tan and Kaveri, 2024; Wei et al., 2023), care (Brass et al., 2024; Chen et al., 2024; Wentzel, 2022), and empathy (Hong et al., 2022; Samavi et al., 2022; Yang and Zhang, 2024) with their students. This development not only benefits learners but also positively impacts the teachers' own wellbeing and professional performance (Akiba and Liang, 2016; Aydin Sunbul and Gordesli, 2021; Corrente et al., 2022; Gore et al., 2017; Hong et al., 2022; Jurkowski and Abramczyk, 2024; Kim et al., 2022; Paliliunas et al., 2024; Reeves et al., 2017; Samavi et al., 2022; Soininen et al., 2023; Yang and Zhang, 2024).

The findings underscore the importance of promoting both resilience and prosociality in teacher self-efficacy. However, this study has some limitations. First, the use of self-reported data may have introduced recall and social desirability biases, despite assurances of anonymity. Additionally, although Harman's single-factor test indicated that common method bias was unlikely to significantly affect the results, the use of self-reported measures may still be subject to bias. Moreover, the standardized nature of the scales limits the ability to capture the contextual specificities of the phenomena; thus, future research should consider using multi-method approaches. Second, the cross-sectional design prevents establishing strong causal inferences, and bidirectional relationships between the analyzed variables cannot be ruled out. Subsequently, the direction of the proposed paths, beyond the outcomes achieved, confines causality to the theoretical realm. Third, the exclusive focus on elementary school teachers in Chile limits the generalizability of the findings, so it would be convenient to explore these associations at other educational levels (e.g., high school) or other cultural contexts where there are likely differences in the administration and educational system. The relationships between resilience, prosociality, and self-efficacy in the study may reflect cultural and institutional features of Chile's centralized primary education system, which shapes how teachers perceive and enact resilience and value prosocial behaviors. Finally, the binary treatment of gender precludes exploration of differences associated with other identities.

Based on these findings, we suggest that both resilience and prosociality should occupy a central role in initial teacher training programs and professional development strategies. It is particularly important to foster formative practices that integrate the development of socioemotional competencies as a transversal axis of pedagogical work—beyond technical or disciplinary mastery. Incorporating specific modules on emotional regulation, prosocial skills, and resilient coping would help prepare future teachers to face diverse, challenging, and changing school environments, strengthening their sense of self-efficacy and professional wellbeing. At the institutional level, these results also support the design and implementation of school policies aimed at creating emotionally sustainable work climates, where mutual support, collaboration, and care are valued as pillars of the teaching profession.

## Data Availability

The datasets presented in this article are not readily available because the data that support the findings of this study are not available because they are confidential data. Requests to access the datasets should be directed to sonia.salvo@ufrontera.cl.

## References

[B1] Abramczyk A. Jurkowski S. (2020). Cooperative learning as an evidence-based teaching strategy: what teachers know, believe, and how they use it. J. Educ. Teach. 46, 296–308. 10.1080/02607476.2020.1733402

[B2] Ainsworth S. Oldfield J. (2019). Quantifying teacher resilience: context matters. Teach. Teach. Educ. 82, 117–128. 10.1016/j.tate.2019.03.01237616319

[B3] Akiba M. Liang G. (2016). Effects of teacher professional learning activities on student achievement growth. J. Educ. Res. 109, 99–11. 10.1080/00220671.2014.92447027409075

[B4] Aldabbagh R. Glazebrook C. Sayal K. Daley D. (2022). Systematic review and meta-analysis of the effectiveness of teacher delivered interventions for externalizing behaviors. J. Behav. Educ. 33, 1–42. 10.1007/s10864-022-09491-4 36093124 PMC9440654

[B5] Alhawsawi H. Alhawsawi S. Sadeck O. (2022). Understanding resilience and coping in a digitally transformed educational environment during COVID-19. J. Furth. High. Educ. 47, 242–254. 10.1080/0309877X.2022.2106124

[B6] Al-Samarraie H. Shamsuddin A. Alzahrani A. I. (2020). A flipped classroom model in higher education: a review of the evidence across disciplines. Educ. Technol. Res. Dev. 68, 1017–1051. 10.1007/s11423-019-09718-840639312

[B7] Aydin Sunbul Z. Gordesli M. (2021). Psychological capital and job satisfaction in public-school teachers: the mediating role of prosocial behaviours. J. Educ. Teach. 47, 147–162. 10.1080/02607476.2021.1877086

[B8] Bagheri A. Saadati M. (2021). Generalized structural equations approach in the of elderly self-rated health. J. Phys. Conf. Ser. 1863, 1–9. 10.1088/1742-6596/1863/1/012041

[B9] Bandura A. (1986). Social Foundations of Thought and Action: A Social Cognitive Theory. Englewood Cliffs, N.J.: Prentice-Hall Inc.

[B10] Bandura A. (1995). “Exercise of personal and collective efficacy in changing societies,” in Self-efficacy in Changing Societies, ed. A. Bandura (New York: Cambridge University Press), 1–45. 10.1017/CBO9780511527692

[B11] Bandura A. (1997). Self-Efficacy: The Exercise of Control. New York, NY: W. H. Freeman and Co.

[B12] Bandura A. (2001). Social cognitive theory: an agentic perspective. Annu. Rev. Psychol. 52, 1–26. 10.1146/annurev.psych.52.1.1 11148297

[B13] Bandura A. (2016). Moral Disengagement. How People Do Harm and Live With Themselves. New York: Worth Publishers.

[B14] Barbaranelli C. Paciello M. Biagioli V. Fida R. Tramontano C. (2019). Positivity and behaviour: the mediating role of self-efficacy in organisational and educational settings. J. Happiness Stud. 20, 707–727. 10.1007/s10902-018-9972-4

[B15] Beltman S. Mansfield C. Price A. (2011). Thriving not just surviving: a review of research on teacher resilience. Educ. Res. Rev. 6, 185–207. 10.1016/j.edurev.2011.09.001

[B16] Bovaird J. A. Koziol N. A. (2012). “Measurement models for ordered-categorical indicators,” in Handbook of Structural Equation Modeling, ed. R. H. Hoyle (New York: The Guilford Press), 495–511.

[B17] Brass N. R. Bergin C. Slaten C. Milarsky T. Mirielli L. Imler M.. (2024). Associations between middle schoolers' adjustment and perceptions of their teachers' social-emotional practices. J. Exp. Educ. 93, 1–22. 10.1080/00220973.2024.2329091

[B18] Bravo-Sanzana M. Miranda R. Oriol X. (2023). Adolescent victimization during COVID-19 lockdowns and its influence on mental health problems in seven countries: the mediation effect of resilience. Int. J. Environ. Res. Public Health 20:1958. 10.3390/ijerph20031958 36767323 PMC9915164

[B19] Browne M. Cudeck R. (1992). Alternative ways of assessing model fit. Sociol. Methods Res. 21, 230–258. 10.1177/0049124192021002005

[B20] Cachón Zagalaz J. López Manrique I. San Pedro Veledo M. B. Zagalaz S. M. L. González González de Mesa C. (2020). The importance of the phoenix bird technique (resilience) in teacher training: CD-RISC scale validation. Sustainability 12:1002. 10.3390/su12031002

[B21] Cann R. Sinnema C. Rodway J. Daly A. J. (2024). What do we know about interventions to improve educator wellbeing? A systematic literature review. J. Educ. Change. 25, 231–227. 10.1007/s10833-023-09490-w

[B22] Caprara G. V. Steca P. Zelli A. Capanna C. (2005). A new scale for measuring adults' prosocialness. Eur. J. Psychol. Assess. 21, 77–89. 10.1027/1015-5759.21.2.77

[B23] Chen L. Zhou Y. F. Nanakida A. (2024). ‘I know I am caring': fostering ethical care awareness and practice among pre-service early childhood teachers. J. Educ. Teach. 51, 1–18. 10.1080/02607476.2024.2447577

[B24] Collie R. J. Shapka J. D. Perry N. E. (2012). School climate and social-emotional learning: predicting teacher stress, job satisfaction, and teaching efficacy. J. Educ. Psychol. 104, 1189–1204. 10.1037/a0029356

[B25] Corrente M. Ferguson K. Bourgeault I. L. (2022). Mental health experiences of teachers: a scoping review. J. Teach. Learn. 16, 23–43. 10.22329/jtl.v16i1.6856

[B26] Costa M. F. B. Cipolla C. M. (2025). Critical soft skills for sustainability in higher education: a multi-phase qualitative study. Sustainability 17:377. su17020377 10.3390/su17020377

[B27] Davis A. N. Clark E. S. Streit C. Kelly R. J. Lardier D. T. (2022). The buffering role of community self-efficacy in the links between family economic stress and young adults' prosocial behaviors and civic engagement. J. Teach. Learn. 183, 527–536. 10.1080/00221325.2022.2094212 35802473

[B28] Di Giunta L. Alessandri G. Gerbino M. Luengo B. P. Zuffianò A. Caprara G. V. (2013). The determinants of scholastic achievement: the contribution of personality traits, self-esteem, and academic self-efficacy. Learn. Individ. Differ. 27, 102–108. 10.1016/j.lindif.2013.07.00621199485

[B29] Diseth A. Danielsen A. G. Samdal O. (2012). A path analysis of basic need support, self-efficacy, achievement goals, life satisfaction and academic achievement level among secondary school students. Educ. Psychol. 32, 335–354. 10.1080/01443410.2012.657159

[B30] Duan S. Bissaker K. Xu Z. (2024). Correlates of teachers' classroom management self-efficacy: a systematic review and meta-analysis. Educ. Psychol. Rev. 36:43. 10.1007/s10648-024-09881-2

[B31] Durrani N. Makhmetova Z. (2025). A multi-layered socio-ecological framework for investigating teacher well-being: key predictors and protective factors. Sustainability 17:90. su17030900 10.3390/su17030900

[B32] Edmonds R. Ochaya A. Sansom N. (2022). The role of resilience processes in education and well-being outcomes for separated children in Uganda: exploring street-connected children's pathways through a resilience-based programme and beyond. Glob. Stud. Child. 12, 14–16. 10.1177/20436106221082677

[B33] Eisenberg N. Guthrie I. K. Cumberland A. Murphy B. C. Shepard S. A. Zhou Q.. (2002). Prosocial development in early adulthood: a longitudinal study. J. Pers. Soc. Psychol. 82, 993–1006. 10.1037/0022-3514.82.6.993 12051585

[B34] Escalante Mateos N. Fernández-Zabala A. Goñi Palacios E. Izar-de-la-Fuente Díaz-de-Cerio I. (2021). School climate and perceived academic performance: direct or resilience-mediated relationship? Sustainability 13:68. 10.3390/su13010068

[B35] Finney S. DiStefano C. (2013). “Structural equation modeling: a second course,” in A Second Course in Structural Equation Modeling, 2nd ed, eds. G. R. Hancock and R. O. Mueller (Charlotte, NC: Information Age Publishing), 439–492.

[B36] Flores-Piñero M. del C. González-Hernández J. Valdivia-Moral P. Á. (2024). Teaching action and altruistic behaviour in physical education classes. predictive analysis applying the 3 × 2 motivational climate model. Revista Española de Pedagogía 82, 651–666. 10.22550/2174-0909.4087

[B37] Gálvez-Nieto J. L. Salvo-Garrido S. Domínguez-Lara S. Polanco-Levicán K. Mieres-Chacaltana M. (2023). Psychometric properties of the teachers' sense of efficacy scale in a sample of chilean public school teachers. Front. Psychol. 14:1272548. 10.3389/fpsyg.2023.1272548 37809312 PMC10556521

[B38] Gómez Tabares A. S. (2018). Prosocialidad. Estado actual de la investigación en Colombia. Revista Colombiana de Ciencias Sociales 10, 188–218. 10.21501/22161201.306529336329

[B39] Gore J. Lloyd A. Smith M. Bowe J. Ellis H. Lubans D. (2017). Effects of professional development on the quality of teaching: results from a randomised controlled trial of quality teaching rounds. Teach. Teach. Educ. 68, 99–113. 10.1016/j.tate.2017.08.007

[B40] Gouveia V. V. Moura H. M. de Santos, L. C. de O. Nascimento A. M. do Guedes, I. de O.. (2018). Escala de autorrelato de trapaça-admissão: evidências de validade fatorial e precisão. Revista Colombiana de Psicología 27, 27–24. 10.15446/rcp.v27n1.64467

[B41] Grotberg E. (1995). A Guide to Promoting Resilience in Children: Strenghening the Human Spirit. The International Resilience Project. Bernard Van Leer. 10.1037/e614412012-002

[B42] Grotberg E. (2001). Resilience programs for children in disaster. Ambulatory Child Health 7, 75–83. 10.1046/j.1467-0658.2001.00114.x

[B43] Grotberg E. (2003). “What is resilience? how do you promote it? how do you use it?” in Resilience for Today: Gaining Strength from Adversity, ed. E. Grotberg (Westport, CT: Praeger Publishers), 1–29. 10.5040/9798216007999.0004

[B44] Hammond Z. (2015). Culturally Responsive Teaching and The Brain: Promoting Authentic Engagement and Rigor Among Culturally and Linguistically Diverse Students. Thousand Oaks, California: Corwin Publishing.

[B45] Hart R. Hart D. (2023). Untying the text: organizational prosociality and kindness. Behav. Sci. 13, 1–13. 10.3390/bs13020186 36829415 PMC9952219

[B46] Hartcher K. Chapman S. Morrison C. (2023). Applying a band-aid or building a bridge: ecological factors and divergent approaches to enhancing teacher wellbeing. Camb. J. Educ. 53, 329–356. 10.1080/0305764X.2022.2155612

[B47] Hong Y. Cai J. Lan R. Wang K. Lian R. Chen L. (2022). Empathy and teachers' fairness behavior: the mediating role of moral obligation and moderating role of social value orientation. PLoS ONE 17:e0268681. 10.1371/journal.pone.0268681 35679271 PMC9182229

[B48] Howard P. (2018). Twenty-first century learning as a radical rethinking of education in the service of life. Educ. Sci. 8, 189. 10.3390/educsci8040189

[B49] Howard P. Corbett M. Burke-Saulnier A. Young D. (2020). Education futures: conservation and change. Paper commissioned for the UNESCO Futures of Education report (forth coming, 2021). UNESCO. Available online at: https://unesdoc.unesco.org/ark:/48223/pf0000374087

[B50] Hu L. T. Bentler P. M. (1999). Cutoff criteria for fit indexes in covariance structure analysis: conventional criteria versus new alternatives. Struct. Equ. Model. 6, 1–55. 10.1080/1070551990954011836787513

[B51] Johnson B. Down B. (2013). Critically re-conceptualising early career teacher resilience. Discourse 34, 703–715. 10.1080/01596306.2013.728365

[B52] Jurkowski S. Abramczyk A. (2024). Collaboration in in-service teacher training - the missing link between empirical evidence and practice? J. Educ. Teach. 50, 550–563. 10.1080/02607476.2024.2327062

[B53] Kay B. (2020). “Supporting living schools through transformative governance and leadership: a vermont experience,” in Living Schools; Transforming Education, eds. P. Howard and C. O'Brien (Winnipeg, Manitoba: ESWB Press), 93–104.

[B54] Kim J. Cillessen A. H. N. (2023). Prospective associations of prosocial behavior and aggression with social preference: moderation by classroom levels of peer-perceived liking and disliking by the teacher. Int. J. Behav. Dev. 47, 423–432. 10.1177/01650254231186327

[B55] Kim L. E. Oxley L. Asbury K. (2022). “My brain feels like a browser with 100 tabs open”: a longitudinal study of teachers' mental health and well-being during the COVID-19 pandemic. British J. Educ. Psychol. 92, 299–318. 10.1111/bjep.12450 34337737 PMC8420299

[B56] Kline R. B. (2005). Principles and Practice of Structural Equation Modeling (2e ed.). New York: Guilford.

[B57] Kraft M. A. Simon S. N. Lyon A. M. (2020). Sustaining a sense of success: the protective role of teacher working conditions during the COVID-19 pandemic. EdWorkingPapers 21, 1–62. 10.26300/35nj-v890

[B58] Li C. H. (2014). The Performance of MLR, USLMV, and WLSMV Estimation in Structural Regression Models with Ordinal Variables. East Lansing: Michigan State University.

[B59] Li C. H. (2016). Confirmatory factor analysis with ordinal data: comparing robust maximum likelihood and diagonally weighted least squares. Behav. Res. Methods 48, 936–949. 10.3758/s13428-015-0619-7 26174714

[B60] Liu Y. Ngai S. (2019). The impact of social capital, self-efficacy, and resilience on the prosocial involvement of adolescents from families with and without economic disadvantages. Child Indic. Res. 12, 1735–1757. 10.1007/s12187-018-9607-7

[B61] Mansfield C. F. Beltman S. Price A. McConney A. (2012). “Don't sweat the small stuff:” understanding teacher resilience at the chalkface. Teach. Teach. Educ. 28, 357–367. 10.1016/j.tate.2011.11.001

[B62] Mieres-Chacaltana M. Salvo-Garrido S. Denegri-Coria M. (2020). Evaluación de la escala de Prosocialidad de Caprara, Steca, Zelli y Capanna en estudiantes universitarios chilenos. Revista Iberoamericana de Diagnóstico y Evaluación – e Avaliação Psicológica 56, 21–32. 10.21865/RIDEP56.3.02

[B63] Mieres-Chacaltana M. Salvo-Garrido S. Dominguez-Lara S. (2025). Modeling the effects of teacher resilience and self-efficacy on prosocialness: implications for sustainable education. Sustainability 17:3874. su17093874 10.3390/su17093874

[B64] Mieres-Chacaltana M. Salvo-Garrido S. Dominguez-Lara S. Gálvez-Nieto J. L. Alarcón-Bañares P. (2023). Psychometric validation of the adult prosocialness behavior scale in a professional teaching context. Behav. Sci. 13:761. bs13090761 10.3390/bs13090761 37754039 PMC10525657

[B65] Mohamadi F. S. Asadzadeh H. Ahadi H. Jomehri F. (2011). Testing bandura's theory in school. Procedia Soc. Behav. Sci. 12, 426–435. 10.1016/j.sbspro.2011.02.053

[B66] Moll Riquelme I. Bagur Pons S. Rosselló Ramon M. R. (2022). Resilience: conceptualization and keys to its promotion in educational centers. Children 9:1183. children9081183 10.3390/children9081183 36010073 PMC9406923

[B67] Mudhar G. Ertesvåg S. K. Pakarinen E. (2023). Patterns of teachers' self-efficacy and attitudes toward inclusive education associated with teacher emotional support, collective teacher efficacy, and collegial collaboration. Eur. J. Spec. Needs Educ. 39, 446–462. 10.1080/08856257.2023.2233297

[B68] Muthén L. K. Muthén B. O. (2017). Mplus User's Guide. Eighth Edition. Los Angeles, CA: Muthén and Muthén.

[B69] Muthén L. K. Muthén B. O. (2019). Mplus (Version 8.4) [Statistical software]. Muthén and Muthén.

[B70] Nielsen K. L. (2023). Why Can the Flipped Classroom Frustrate Students? Experiences from an engineering mathematics course. Educ. Sci. 13:396. educsci13040396 10.3390/educsci13040396

[B71] Paliliunas D. Burke R. Taylor S. Belisle J. Frizell C. Sickman E. (2024). A preliminary analysis of a prosocial intervention to support teachers and staff implementing behavioral interventions in a specialized school setting. Behav. Anal. Pract. 17, 1191–1197. 10.1007/s40617-024-01005-0 39790921 PMC11707119

[B72] Podsakoff P. M. MacKenzie S. B. Lee J. Y. Podsakoff N. P. (2003). Common method biases in behavioral research: a critical review of the literature and recommended remedies. J. Appl. Psychol. 88, 879–903. 10.1037/0021-9010.88.5.879 14516251

[B73] Pozo-Rico T. Poveda R. Gutiérrez-Fresneda R. Castejón J.-L. Gilar-Corbi R. (2023). Revamping teacher training for challenging times: teachers' well-being, resilience, emotional intelligence, and innovative methodologies as key teaching competencies. Psychol. Res. Behav. Manag. 16, 1–18. 10.2147/PRBM.S382572 36636290 PMC9830420

[B74] Reeves P. M. Hung Pun W. Sun Chung K. (2017). Influence of teacher collaboration on job satisfaction and student achievement. Teach. Teach. Educ. 67, 227–236. 10.1016/j.tate.2017.06.016

[B75] Saavedra E. Villalta M. (2008). Medición de las características resilientes, un estudio comparativo en personas entre 15 y 65 Años. Liberabit 14, 31–4.

[B76] Salinas-Falquez S. Roman-Lorente C. Buzica M. Álvarez J. Gutiérrez N. Trigueros R. (2022). Teachers' mental health and their involvement in educational inclusion. Behav. Sci. 12:261. bs12080261 10.3390/bs12080261 36004832 PMC9405262

[B77] Salvo S. Bravo-Sanzana M. Miranda-Vargas H. Fóres A. Mieres-Chacaltana M. (2017). ¿La promoción de la resiliencia en la escuela puede contribuir con la política pública de salud? Salud Publica de Mexico 59, 214–215. 10.21149/8328 28902307

[B78] Salvo-Garrido S. Polanco-Levicán K. Dominguez-Lara S. Mieres-Chacaltana M. Gálvez-Nieto J. L. (2023). Psychometric properties of the SV-RES60 resilience scale in a sample of chilean elementary school teachers. Behav. Sci. 13:781. bs13090781 10.3390/bs13090781 37754059 PMC10525169

[B79] Salvo-Garrido S. Polanco-Levicán K. Dominguez-Lara S. Mieres-Chacaltana M. Gálvez-Nieto J. L. (2024). Relationships between resilience and self-efficacy in the prosocial behavior of chilean elementary school teachers. Behav. Sci. 14:678. bs14080678 10.3390/bs14080678 39199075 PMC11352048

[B80] Samavi A. Hajializadeh K. Javdan M. Farshad M. R. (2022). Psychometric validation of teacher empathy scale: measurement invariance in gender. Front. Psychol. 13:1042993. 10.3389/fpsyg.2022.1042993 36507024 PMC9733426

[B81] Satorra A. Bentler P. M. (1994). “Corrections to test statistics and standard errors in covariance structure analysis,” in Latent variables analysis: Applications for Developmental Research, eds. A. von Eye and C. C. Clogg (Thousand Oaks, CA: Sage Publications, Inc), 399–419.

[B82] Schumacher R. Lomax R. (1996). A Beginner's Guide to Structural Equation Modeling. New York: Lawrence Erlbaum Associates.

[B83] Silveira S. Hecht M. Adli M. Voelkle M. C. Singer T. (2022). Exploring the structure and interrelations of time-stable psychological resilience, psychological vulnerability, and social cohesion. Front. Psychiatry 13:804763. 10.3389/fpsyt.2022.804763 35360131 PMC8963374

[B84] Sisto A. Vicinanza F. Campanozzi L. L. Ricci G. Tartaglini D. Tambone V. (2019). Towards a transversal definition of psychological resilience: a literature review. Medicina 55:745. medicina55110745 10.3390/medicina55110745 31744109 PMC6915594

[B85] Soininen V. Pakarinen E. Lerkkanen M. K. (2023). Reciprocal associations among teacher–child interactions, teachers' work engagement, and children's social competence. J. Appl. Dev. Psychol. 85:101508. 10.1016/j.appdev.2022.101508

[B86] Steiner E. D. Woo A. (2021). Job-Related Stress Threatens the Teacher Supply: Key Findings from the 2021 State of the U.S. Teacher Survey. RAND Corporation. Available online at: https://www.rand.org/pubs/research_reports/RRA1108-1.html (Accessed January 15, 2025).

[B87] Tan E. Kaveri G. (2024). Building prosocial behaviours: examining the possibilities of social stories in early childhood classroom settings. J. Early Child. Res. 22, 458–447. 10.1177/1476718X241227044

[B88] Trevors J. T. Saier M. H. (2010). Education for humanity. Water Air Soil Pollut. 206, 1–2. 10.1007/s11270-009-0269-4 22247577 PMC3252885

[B89] Tschannen-Moran M. Woolfolk Hoy A. (2001). Teacher efficacy: capturing an elusive construct. Teach. Teach. Educ. 17, 783–805. 10.1016/S0742-051X(01)00036-1

[B90] UNESCO (2024). Informe de seguimiento de la educación en el mundo 2023: Tecnologí*a en la educación: ¿Una herramienta en los términos de quién?* Paris: UNESCO.

[B91] Ungar M. (2012). “Social ecologies and their contribution to resilience,” in The Social Ecology of Resilience, ed. M. Ungar (New York: Springer), 13–31. 10.1007/978-1-4614-0586-3

[B92] United Nations (2016). Naciones Unidas. Asamblea General de las Naciones Unidas. Resoluciones y Decisiones Aprobadas Por La Asamblea General Durante Su Septuagésimo Período de Sesiones. Volumen I: Resoluciones 15 de Septiembre a 23 de Diciembre de 2015. Asamblea General Documentos Oficiales Septuagésimo Período de Sesiones. Suplemento. Available online at: https://www.un.org/es/ga/70/resolutions.shtml

[B93] United Nations (2022). Human Development Report 2021/2022. Uncertain times, unsettled lives. Shaping our future in a transforming world. United Nations Development Programme (UNDP). Available online at: https://www.undp.org/egypt/publications/human-development-report-2021-22-uncertain-times-unsettled-lives-shaping-our-future-transforming-world (Accessed February 3, 2025).

[B94] Villalta M. Saavedra E. (2011). Cultura escolar, prácticas de enseñanza y resiliencia en alumnos y profesores de contextos sociales vulnerables. Universitas Psychologica 11, 67–78. 10.11144/Javeriana.upsy11-1.cepe

[B95] Wang J. (2024). Research on the flipped classroom + learning community approach and its effectiveness evaluation—taking college german teaching as a case study. Sustainability 16:7719. su16177719 10.3390/su16177719

[B96] Wang J. Wang X. (2020). Structural Equation Modeling. Applications Using Mplus. Hoboken NJ: Wiley. 10.1002/9781119422730

[B97] Wang X. Gao Y. Wang Q. Zhang P. (2024). Relationships between self-efficacy and teachers' well-being in middle school English teachers: the mediating role of teaching satisfaction and resilience. Behav. Sci. 14:629. 10.3390/bs14080629 39199025 PMC11351107

[B98] Wang X. L. Lo L. N. K. (2022). Development of resilience among Chinese rural teachers: a social ecological perspective. Teach. Teach. 28, 533–554. 10.1080/13540602.2022.2062743

[B99] Wei B. Zhang X. Xiao X. Li Y. (2023). The effect of different types of social norms on children's sharing behavior: the roles of parents, teachers, and peers. Soc. Dev. 32, 1–19. 10.1111/sode.1266327409075

[B100] Wentzel K. R. (2022). Does anybody care? Conceptualization and measurement within the contexts of teacher-student and peer relationships. Educ. Psychol. Rev. 34, 1919–1954. 10.1007/s10648-022-09702-4

[B101] Xiang X. P. Wang J. Tian G. X. Ungar M. Han L. L. (2023). Risk, resilience, and Chinese youth's psychosocial adjustment: the role of social services. J. Soc. Serv. Res. 49, 147–116. 10.1080/01488376.2023.219827939645377

[B102] Yang S. L. Wang W. R. (2022). The role of academic resilience, motivational intensity and their relationship in EFL learners' academic achievement. Front. Psychol. 12:823537. 10.3389/fpsyg.2021.823537 35153940 PMC8826434

[B103] Yang T. Zhang Y. (2024). Prosocial development in early childhood: strategies of teachers and parents during the COVID-19 pandemic in China. Early Child Dev. Care 194, 1–16. 10.1080/03004430.2024.2383916

